# Serum myoglobin modulates kidney injury via inducing ferroptosis after exertional heatstroke

**DOI:** 10.2478/jtim-2023-0092

**Published:** 2023-07-05

**Authors:** Yingyi Luan, Enping Huang, Jiajia Huang, Zhenjia Yang, Zhipeng Zhou, Yan Liu, Conglin Wang, Ming Wu

**Affiliations:** Department of Infection and Critical Care Medicine, Shenzhen Second People's Hospital & First Affiliated Hospital of Shenzhen University, Health Science Center, Shenzhen 518035, China; Department of Central Laboratory, Beijing Obstetrics and Gynecology Hospital, Capital Medical University; Beijing Maternal and Child Health Care Hospital Beijing 100026, China; Department of Forensic Medicine, Southern Medical University, Guangzhou 510515, Guangdong Province, China; Shantou University Medical College, Shantou 515041, Guangdong Province, China; Department of critical care medicine, General Hospital of Southern Theatre Command of PLA, Guangzhou 510010, Guangdong Province, China

**Keywords:** myoglobin, exertional heatstroke, acute kidney injury, ferroptosis, endoplasmic, reticulum stress, baicalein

## Abstract

**Background and Objectives:**

Myoglobin released by rhabdomyolysis (RM) is considered to be involved in pathogenesis of kidney disease caused by crush injury, but whether high level of serum myoglobin predisposes patients to acute kidney injury (AKI) and its molecular mechanisms are still unclear in exertional heatstroke (EHS). We aimed to determine the association and potential mechanism of myoglobin and AKI, and further investigate the targeted therapeutic agents for myoglobinemia.

**Methods:**

Serum myoglobin concentrations in patients with EHS were measured at admission, 24 h and 48 h after admission and discharge. The risk of AKI at 48 h was the primary outcome; the secondary outcome was composite outcome events with myoglobin levels and AKI at discharge and death at 90 days. In experimental studies, we further investigated the mechanisms of human kidney proximal tubular (HK-2) cells that were exposed to human myoglobin under heat stress conditions and the effect of baicalein.

**Results:**

Our measurements showed that the highest myoglobin quartile (*vs*. the lowest) had an adjusted odds ratio (OR) of 18.95 (95% confidence interval [CI], 6.00–59.83) for AKI and that the OR (*vs*. quartile 2) was 7.92 (95% CI, 1.62-38.89) for the secondary outcome. The survival rate of HK-2 cells treated with myoglobin under heat stress was significantly decreased, and the production of Fe2+ and reactive oxygen species (ROS) was markedly increased, accompanied by changes in ferroptosis proteins, including increased p53, decreased SLC7A11 and GPX4, and alterations in endoplasmic reticulum stress (ERS) marker proteins. Treatment with baicalein attenuated HK-2 cell ferroptosis induced by myoglobin under heat stress through inhibition of ERS.

**Conclusions:**

High myoglobin was associated with AKI in the EHS, and its mechanisms involved ERS-associated ferroptosis. Baicalein may be a potential therapeutic drug for the treatment of AKI in patients with high myoglobin induced by rhabdomyolysis following EHS.

## Introduction

A record-breaking heat wave attacked the Pacific Northwest and western Canada in 2021. Approximately, more than 600 people died than that would have been typical in Oregon and Washington in late June. In Canada, at least 486 unexpected deaths were reported during a five-day period, which was nearly three times higher than usual. With global warming, a high-temperature climate has a disastrous impact on public health. Heatstroke is a clinical syndrome characterized by an imbalance in body temperature regulation, disorder of water and electrolytes, and impairment of nervous system function. Exertional heatstroke (EHS) has a poorer prognosis than classical heatstroke, which often combines rhabdomyolysis (RM),^[1]^ acute kidney injury (AKI),^[2]^ acute hepatic injury (AHI),^[3]^ diffuse intravascular coagulation (DIC),^[4]^ and even multiple organ dysfunction syndrome (MODS), with 40%–70% mortality and 30% disability.^[5,6]^

EHS is significantly different from massive crush syndrome, due to involving heat injury factors, the myoglobin released from rhabdomyolysis caused by EHS increases gradually, usually without serious trauma and extrusion, but hypermyoglobinemia can aggravate the injury of other organs, and its treatment methods are also different.^[7,8]^ Studies have shown that serum myoglobin exceeds the renal threshold of 0.5–1.5 mg/dL; hence myoglobinuria will occur. When serum myoglobin reaches 100 mg/dL due to severe trauma, extrusion, and other factors, reddish brown or tan urine can be seen, accompanied by renal tubular cell apoptosis,^[9,10]^ little known of what does serum myoglobin 1.5 mg/dL effect renal tubule cells. In our previous study, we found that serum myoglobin is associated with AKI, especially in patients with serum myoglobin exceeded 100 mg/dL.^[11]^ Based on the clinical practice required, it is urgent to further study the dose-effects relationship and molecular mechanism of myoglobin and AKI, and possible preventive drugs for myoglobinemia caused by EHS and AKI.

Recent studies have shown that the direct injury of lipid peroxidation in proximal tubules induced by divalent iron ions produced by myoglobin metabolism through the Fenton reaction may be a cardinal mechanism of kidney injury induced by RM,^[12]^ in which ferritin heavy chain plays an important role in acute renal tubular injury. In the ferroptosis model induced by RM, various small molecule inhibitors (ferrostatin-1 and ferrostatin) to prevent lipid peroxidation could effectively inhibit cell necrosis induced by hydroxyquinoline and ammonium ferrous sulfate.^[13]^ Although the conventional activities of p53 such as cell cycle arrest, senescence, and apoptosis are well accepted as the major checkpoints in stress responses, accumulating evidence implicates the importance of other tumor suppression mechanisms. Unlike apoptotic cell death, activation of p53 alone is not sufficient to induce ferroptosis directly, instead, through its metabolic targets. p53 is able to modulate the ferroptosis response in the presence of ferroptosis inducers such as GPX4 inhibitors or high levels of reactive oxygen species(ROS).^[14,15]^ It is unclear whether myoglobin has an effect on ferroptosis in kidney tubular cells, *i.e*., the cells most affected in heatstroke complicated with AKI.

Therefore, in the present study, we designed a retrospective study to investigate the dynamic change of serum myoglobin on admission, 24 h, 48 h after admission, and discharge, and outcomes of patients with EHS who admitted to the intensive care unit (ICU) in a tertiary-care teaching hospital in southern China. Then we also used experimental models to investigate the effects and potential mechanism of myoglobin to HK-2 cell survival during heat stress and the potential targeted therapeutic agents for myoglobinemia.

## Methods

### Study design and participants

A retrospective cohort study was performed in the ICU of the General Hospital of Southern Theatre Command of Peoples Liberation Army from January 2008 to June 2019. The inclusion criterion was as follows: patients with “exertional heatstroke” caused by strenuous exercise performed in a high-temperature and high-humidity environment. The diagnostic criteria of exertional heatstroke were as follows: exposure to a high temperature or high humidity and a history of strenuous exercise; a clinical syndrome causing an excessively high body temperature (central temperature higher than 40℃); nervous system dysfunction (including delirium, cognitive impairment, coma, *etc*.); or systemic organ dysfunction. The exclusion criteria were as follows: 1) death or discharge within 24 h after admission; 2) incomplete data regarding key indicators; 3) incomplete outcome evaluation data obtained *via* telephone follow-up; and 4) a previous history of organ dysfunction, such as chronic kidney disease. AKI was defined as KDIGO standard.^[16]^ The primary outcome was the relationships between myoglobin levels and risk of AKI; the secondary outcome was composite outcome events with myoglobin levels and risk of AKI at discharge and death at 90 days.

The cause of heatstroke is strenuous outdoor training in southern China, and all patients are treated with cooling immediately on site and continue to cool till 39℃ (preferably 38.5℃ to 38.0℃) after admission. All patients received basic life support treatment according to their condition and were provided comprehensive treatment, including brain protection and mechanical ventilation. Moreover, appropriate volume management was performed for patients with AKI, and renal replacement therapy such as continuous renal replacement therapy (CRRT) was initiated, if necessary.

The study was approved by the Research Ethics Committee of the General Hospital of Southern Theatre Command of PLA (HE-2020-09). In view of the retrospective study design and depersonalization of data, the Ethics Committee agreed to waive the requirement for patient written informed consent but required that the patients be informed of the study details during a telephone follow-up.

### Heat Stress Cell Model

HK-2 cells (National Collection of Authenticated Cell Cultures, CBP60447) were cultured in Dulbecco’s Modified Eagle Medium (DMEM, Lonza, Basel, Switzerland), and then HK-2 cells were treated with human myoglobin (882.35 nmol/L, cell assay concentration based on myoglobin renal threshold 1.5 mg/dL) (All from Abcam, Cambridge, MA, USA) at 43℃ for 2 h and rewarmed at 37℃ for different time points (0 h, 1 h, 3 h, 6 h, and 12 h). All cells are cultured at 37°C with 5% CO^2^ (v/v). All media were supplemented with 10% heat-inactivated fetal bovine serum, penicillin (100 U/mL), streptomycin (100 U/mL), and 1× GlutaMAX Supplement (Thermo Fisher Scientific, Waltham, MA, USA).

### p53 depletion

Small interference RNA (siRNA) sequence that target p53 was designed by Shanghai GenePharma Co. Ltd (Shanghai, China), as shown below: siP53 (5¢-CGAUGGUGUUACUUCCUGATT-3¢). siRNA transfection was performed according to the instruction of Lipofectamine 3000 Transfection Reagent Kit from Invitrogen (Thermo Fisher Scientific). HK-2 cells seeded on a 6-well plate were grown to 70%–90% confluence. Lipofectamine 3000 reagent and 5 μmol/L siRNA or control siRNA were mixed with Opti-MEM (Thermo Fisher Scientific, Waltham, MA, USA). The mixed solution was vortexed for 2 s to 3 s, and then incubated for 20 min at room temperature prior to incubation with cells for 6 h. Thereafter, the siRNA/Lipofectamine 3000 complex medium was replaced with the same volume of regular fetal bovine serum-supplemented culture medium. Then, 48 h after transfection, the depletion of p53 was confirmed by Western blotting, and cells were used in subsequent experiments.

### CCK8

Human HK-2 cells were plated into 96-well flat bottom plates at 2×10^5^ cells/well, and incubated with myoglobin (882.35 nmol/ L) and heat stress treatment in medium containing 10% fetal calf serum (FCS) at 37 °C in 5% CO^2^ in humidified air for 24 h. Thereafter, 10 μL CCK-8 solution (Dojindo, Nagasaki, Japan) was added to each well, and the optical density of T-cell proliferative activity was measured by the use of a microplate reader.

### Western blot

Western blotting was performed to determine the expression of inositol-requiring protein 1 (IRE1) p-IRE1/IRE1, eIF2, p-eIF2, PERK, p-PERK, transcription factor 4 (ATF-4), transcription factor 6 (ATF-6), C/EBP-homologous protein (CHOP), p53, cystine glutamate transporter (SLC7A11), and glutathione peroxidase (GPX4) in HK-2 cells. Protein concentrations were measured using a bicinchoninic acid (BCA) protein assay kit (Thermo Scientific, Grand Island, NY, USA). Proteins of HK-2 cells were separated by SDS PAGE and then transferred electrophoretically onto polyvinylidenedifluoride (PVDF) membranes. The membranes were probed with anti-p-IRE1/IRE1 antibody, anti-ATF-4/6 antibody anti-CHOP antibody, anti-P53 antibody, anti-SLC7A11 antibody, anti-GPX4 antibody (Abcam, Cambridge, MA, USA), and polyclonal anti-actin antibodies (Santa Cruz Biotechnology, Dallas, USA). Protein bands were detected using an Odyssey System from LI-COR Biosciences, USA.

### Measurement of ROS and Fe2+

The level of intracellular ROS was determined by the oxidation-sensitive fluorescent probe DCFH-DA with flow cytometry. The level of Fe^2+^ was detected by an iron assay kit purchased from Nanjing Jiancheng Bioengineering Institute and tested at a wavelength of 520 nm.

### Statistical analysis

The continuous variables conforming to a normal distribution are expressed as Mean ± SD. Count data and ordinal data are represented as medians and interquartile ranges (IQRs). Count data were compared using multiple independent samples nonparametric Kruskal–Wallis *H* tests, and measurement data for intergroup comparisons were analyzed using nonparametric Mann–Whitney U tests. Data sets were examined by one-way ANOVA. Statistical analyses were performed using SPSS Windows version 23.0 (SPSS Inc., Chicago, IL, USA), Empower (R) (http://www.empowerstats.com, X&Y solutions, Inc., Boston, MA, USA), and R (http://www.R-project.org) software. *P* values (two-tailed) less than 0.05 were considered statistically significant.

## Results

### Outcomes and baseline characteristics of serum biomarkers of RM in patients who underwent EHS stratified according to the incidence of AKI

A total of 187 patients were included, and all of them were males. Of these, 82 (43.9%) patients had AKI at admission, and stage 1, stage 2, and stage 3 were 62/82 (75.6%), 11/82 (13.4%), and 9/82 (11.0%), respectively. 21 (12.4%) patients had AKI at discharge, and 10/21 (47.6%), 6/21 (28.6%), and 5/21 (23.8%) patients had stage 1, stage 2, and stage 3 AKI, respectively. The 90-day mortality was higher in AKI than in non-AKI (26.8% *vs*. 1.0%, *P <* 0.001). The levels of myoglobin (Mb) and creatine kinase (CK) at admission, 24 h, 48 h, and discharge were higher in patients with AKI than in non-AKI patients (Table 1).

**Table 1:** Clinical serum biomarker of rhabdomyolysis and outcomes of patients who underwent exertional heatstroke, stratified according to incidence of acute kidney injury

Variables	Non-AKI (n = 106)	AKI (n = 82)	*P* value
Age, years	23.6±7.9	26.1±11.0	0.064
Males	106 (100)	82 (100)	1.000
MB (ng/mL)			
admission	186.2	1000.0	<0.001
	(60.5-582.0)	(469.9–1000.0)	
24 hours	70.2 (25.3-217.4)	461.8 (142.0-1000.0)	<0.001
48 hours	61.0	485.0	<0.001
	(33.5-298.1)	(188.8-1000.0)	
discharge	38.5	516.0	<0.001
	(21.0-76.5)	(90.0-1000.0)	
CK(U/L)			
admission	792.0	1146.5	0.018
	(249.0-2111.5)	(519.8-3684.2)	
24 hours	815.0	2083.0	<0.001
	(248.0-2252.0)	(850.2-4913.5)	
48 hours	779.0 (249.0-2630.0)	1892.0 (629.0-4644.0)	0.005
discharge	176.0	281.0	0.067
	(93.0-615.0)	(112.5-1572.5)	
CK-Mb (ng/mL)			
admission	32.5	45.0	0.005
	(22.0–59.8)	(29.0–101.5)	
24 hours	32.5	57.0	<0.001
	(17.2–62.0)	(32.0–104.5)	
48 hours	28.0	40.0	0.281
	(21.0–60.0)	(21.0–73.0)	
discharge	13.0	16.0	<0.001
	(8.8–19.0)	(7.7–30.5)	
90-day mortality	1 (0.9)	22 (26.8)	<0.001

Data are presented as Mean±SD, *_n_* (%) or median (IQR).

### Association of myoglobin and AKI and 90-day mortality following EHS

**Figure 1:**
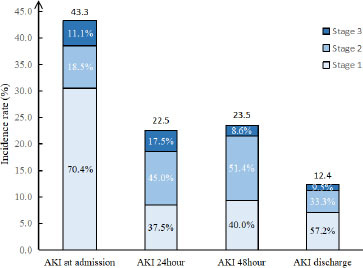
The distributions of AKI and AKI stage at different time. AKI: acute kidney injury.

**Figure 2:**
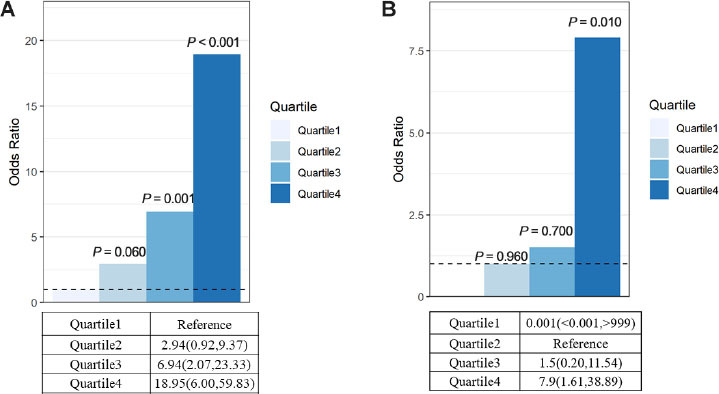
Primary outcome and secondary outcome. (A) Primary outcome: myoglobin levels and risk of AKI. This model was adjusted for age. Quartile 1 was the reference (R) group. Quartile1<127, Quartile2: 127–468.9, Quartile3: 468.9–1000, Quartile4≥1000 Quartile1 5/39 (12.82%), Quartile2 12/40 (30.0%), Quartile3 14/28 (50.0%), Quartile4 38/52 (73.08%). (B) Secondary outcome: myoglobin levels and risk of AKI at discharge and death at 90 d. Quartile1 0 (0.0%), Quartile2 3/40 (7.5%), Quartile3 3/29 (10.3%), Quartile4 18/52 (34.6%). AKI: acute kidney injury.

The characteristics of the patients, stratified according to quartiles of myoglobin level, are shown in Figure 1. For rhabdomyolysis after heatstroke, the incidence of AKI was 73.08% in the highest myoglobin quartile (≥ 1000 ng/mL) and 12.82% in the lowest quartile (< 127 ng/mL), which yielded an adjusted odds ratio of AKI that was 18.95 (95% confidence interval [CI], 6.00–59.83) times as high in the highest quartile as in the lowest quartile (Figure 1). The myoglobin level was also strongly associated with the combined outcome of acute kidney injury and death at 90 d (adjusted odds ratio, 7.9; 95% CI, 1.61–38.89) (Figure 2).

### Myoglobin is associated with HK-2 cell ferroptosis in a heat stress model

**Figure 3:**
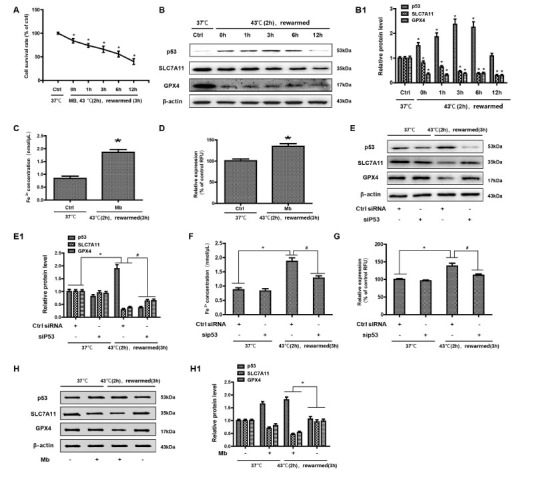
Myoglobin is associated with HK-2 cell ferroptosis in a heat stress model. (A) Human HK-2 cells were treated with myoglobin at concentrations of 882.35nmol/L at 43℃ **for 2 h and rewarmed at 37**℃ **at different time points (0 h, 1 h, 3 h, 6 h, and 12 h). The effect of heat stress and myoglobin on the survival of HK-2 cells and the cell survival rate were examined. (B) Western blot and quantitative analyses (B1) were performed to evaluate the expression of p53, SLC7A11, and GPX4. (C and D) Intracellular Fe^2+^ and ROS in HK-2 cells were detected. (E, E1) HK-2 cells were transfected with control (ctrl) siRNA or siP53 for 48 h, treated with myoglobin at a concentration of 882.35nmol/L at 43**℃ **for 2 h, and rewarmed at 37**℃ **for 3 h. (F and G) HK-2 cells were transfected with control (ctrl) siRNA or siP53 for 48 h, treated with myoglobin at a concentration of 882.35 nmol/L at 43**℃ **for 2 h, and rewarmed at 37**℃ for 3 h. Intracellular Fe^2+^ and ROS in HK-2 cells were detected. (H and H1) Western blot and quantitative analyses were performed evaluate the expression of p53, SLC7A11, and GPX4. Western blot and quantitative analyses were performed evaluate the expression of p53, SLC7A11, and GPX4; The values are the mean ± SD from triplicate independent experiments with statistical significance: *P < 0.05 *vs*. the control group; ^#^*P* < 0.05 *vs*. the scrambled+rewarmed group.

To determine the role of myoglobin in the mechanism of human kidney proximal tubular (HK-2) cell function, HK-2 cells were treated with myoglobin at concentrations of 882.35nmol/L at 43℃ for 2 h and rewarmed at 37℃ at different time points (0 h, 1 h, 3 h, 6 h, and 12 h). First, we examined the effect of heat shock and myoglobin on the survival of HK-2 cells, and the cell survival rate began to decline after 0 h of rewarming (Figure 3A, *P* < 0.01). Simultaneously, with the extension of rewarming time, the cell survival rate decreased significantly; after 12 h of rewarming, the cell survival rate decreased to less than 50%. We further determined whether ferroptosis was involved in severe heatstroke complicated with AKI. Both of SLC7A11 and GPX4 expression in HK-2 cells were detected by Western blot. We found that compared with the control group at 37℃, the expression of p53 was significantly increased in response to myoglobin after heat stress for 0 h. With the extension of rewarming time, the expression increased and then decreased and reached its highest level at 3 h of rewarming (Figure 3B, *P* < 0.01). As shown in Figure 1C, the expression levels of SLC7A11 and GPX4 were significantly decreased after heat stress and myoglobin treatment for 0 h, and the expression continuously decreased with the extension of rewarming time. Furthermore, the change in intracellular iron content is a very important indicator of ferroptosis. We evaluated HK-2 cell ferroptosis by detecting the intracellular iron content and found that intracellular Fe^2+^ was markedly increased at 3 h of rewarming after heat stress and myoglobin treatment (Figure 3C, *P* < 0.01). Additionally, the level of ROS was significantly increased at 3 h of rewarming in the heat stress- and myoglobin-treated cell model, suggesting that heat shock and myoglobin could induce the production of ROS and activate oxidative stress in HK-2 cells (Figure 3D, *P* < 0.01). Then, silencing of the p53 gene was used *via* a siRNA approach. SLC7A11 and GPX4 expression in HK-2 cells was significantly decreased at 3 h of rewarming in response to myoglobin under heat stress, and these effects were prevented by p53 gene silencing in HK-2 cells (Figure 3E, *P* < 0.01). After p53 gene knockdown, intracellular iron content and ROS levels were significantly decreased compared to that of control-siRNA group under heat stress (Figure 3F and G, *P* < 0.01). Further, the additional groups for myoglobin (at concentrations of 882.35 nmol/L at 37℃ for 3 h) alone were provided in Figure 3H. Taken together, our data show that myoglobin appears to be related to human HK-2 cell ferroptosis under heat stress by affecting SLC7A11 and GPX4 expression, intracellular iron content, and the production of ROS.

### Myoglobin modulates HK-2 cell ferroptosis involving ERS after heat stress

**Figure 4:**
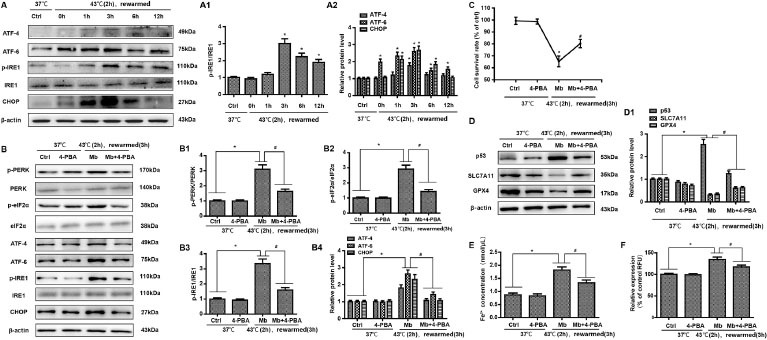
Myoglobin modulates HK-2 cell ferroptosis involving endoplasmic reticulum stress after heat stress. (A, A1) Human HK-2 cells were treated with myoglobin at concentrations of 882.35nmol/L at 43℃ **for 2 h and rewarmed at 37**℃ **at different time points (0 h, 1 h, 3 h, 6 h, and 12 h). The expression levels of p-IRE1/IRE1, ATF-4, ATF-6, and CHOP were measured by Western blot. (B, B1, B2, B3, B4) Human HK-2 cells were pretreated with 4-BPA, treated with myoglobin at concentrations of 882.35nmol/L at 43**℃ **for 2 h, and rewarmed at 37**℃ **for 3 h. The expression levels of p-PERR/PERK, p-eIF2, p-IRE1/IRE1, ATF-4, ATF-6, and CHOP were measured by Western blot. (C) Human HK-2 cells were pretreated with 4-BPA, treated with myoglobin at concentrations of 882.35nmol/L at 43**℃ **for 2 h, and rewarmed at 37**℃ for 3 h. The cell survival rate of HK-2 cells was examined by CCK8. (D) Western blot and quantitative analyses (D1) were performed to evaluate the expression of P53, SLC7A11, and GPX4. (E and F) Intracellular Fe^2+^ and ROS in HK-2 cells were detected. The values are the mean ± SD from triplicate independent experiments with statistical significance: *P < 0.05 the control group; ^#^*P* < 0.05 *vs*. the Mb group.

We further analyzed the expression of ERS-related proteins by Western blot, mainly detected the three classical pathways of ERS, and analyzed the expression of ATF-4, CHOP, ATF-6, IRE1, and p-IRE1. HK-2 cells treated with myoglobin and heat stress showed elevated expression of phosphorylation of PKR-like ER kinase (PERK) p-IRE1/IRE1, ATF-4, ATF-6 and CHOP, and with the extension of rewarming time, their expression first increased and then decreased (Figure 4A, *P* < 0.01). To confirm this, we used an ERS inhibitor (4-BPA) to treat HK-2 cells. There was a significant increase in the expression of p-PERR/PERK, p-eIF2, p-IRE1/IRE1, ATF-4, ATF-6, and CHOP at 3 h of rewarming in response to myoglobin under heat stress; thus, pretreatment with 4-BPA markedly decreased the expression of p-PERR/PERK, p-eIF2, p-IRE1/IRE1, ATF-4, ATF-6, and CHOP in the heat stress and myoglobin groups (Figure 4B, *P* < 0.01). Together, these data reveal the important role of myoglobin in aggravating the ERS response in HK-2 cells during heatstroke. Further, we found that the cell survival rate was significantly decreased after heat stress and myoglobin treatment rewarming for 3 h, and the cell survival rate was markedly increased in the 4-BPA + myoglobin+ heat stress group (Figure 4C, *P* < 0.01). Western blot analysis showed that inhibition of ERS using 4-BPA decreased p53 activation and enhanced SLC7A11 and GPX4 expression in HK-2 cells after heat stress and myoglobin treatment (Figure 4D, *P* < 0.01). Notably, intracellular Fe^2+^ and ROS were significantly decreased in response to heat stress and myoglobin combined with 4-BPA treatment rewarming for 3 h (Figure 4 E and F, *P* < 0.01), indicating that inhibiting ERS could suppress the accumulation of iron and ROS production induced by myoglobin under heat stress.

### Baicalein protects HK-2 cell ferroptosis induced by myoglobin under heat stress by inhibiting ERS

**Figure 5:**
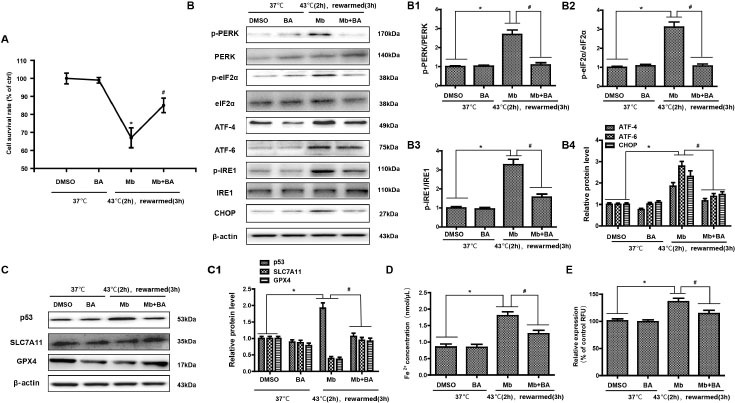
Baicalein protects HK-2 cell ferroptosis induced by myoglobin under heat stress by inhibiting endoplasmic reticulum stress. (A) Human HK-2 cells were treated with myoglobin at a concentration of 882.35nmol/L and baicalein (Selleck Chemicals, USA) at a concentration of 20 μmol/L at 43℃ **for 2 h and rewarmed at 37**℃ for 3 h. HK-2 cells were treated with DMSO as control. The cell survival rate of HK-2 cells was examined by CCK8. (B, B1, B2, B3, B4) Total protein was stained, and p-PERR/PERK, p-eIF2, p-IRE1/IRE1, ATF-4, ATF-6, and CHOP were analyzed by Western blotting. (C) Western blot and quantitative analyses (C1) were performed to evaluate the expression of p53, SLC7A11, and GPX4. (D and E) Intracellular Fe^2+^ and ROS in HK-2 cells were detected. The values are the mean ± SD from triplicate independent experiments with statistical significance: *P < 0.05 the DMSO group; ^#^*P* < 0.05 *vs*. the Mb group.

Recent studies have reported that ERS and ferroptosis are involved in AKI induced by heat stress combined with myoglobin, and ERS inhibitors can effectively inhibit ferroptosis and alleviate heat stress and myoglobin-mediated kidney injury. Therefore, we considered whether there are clinical drugs that can inhibit ferroptosis by regulating ERS to alleviate rhabdomyolysis-induced AKI after EHS. In the present study, as expected, treatment with baicalein increased the cell survival rate after heat stress and myoglobin treatment rewarming for 3 h (Figure 5A, *P* < 0.01), suggesting the protective role of baicalein in AKI under heat stress. Furthermore, baicalein inhibited the activation of ERS signaling, as demonstrated by decreased expression of p-PERK/PERK, p-eIF2, p-IRE1/IRE1, ATF-4, ATF-6, and CHOP in the heat stress and myoglobin treatment rewarming for 3 h group (Figure 5B, *P* < 0.01). Simultaneously, baicalein alleviated ferroptosis in HK-2 cells, as shown by decreased p53 activation and elevated SLC7A11 and GPX4 expression (Figure 5C, *P* < 0.01). Notably, intracellular Fe^2+^ and ROS were markedly decreased in response to heat stress and myoglobin combined with baicalein treatment rewarming for 3 h (Figure 5 D and E, *P* < 0.01), indicating that baicalein could inhibit the accumulation of iron and ROS production induced by myoglobin under heat stress.

## Discussion

This retrospective cohort study showed that myoglobin was associated with subsequent AKI following EHS. Concurrently, we obtained experimental evidence that myoglobin might be directly involved in the pathogenesis of heat stress in HK-2 cells by modulating cellular ferroptosis and increasing endoplasmic reticulum stress. Inhibiting endoplasmic reticulum stress attenuated ferroptosis in HK-2 cells exposed to myoglobin under heat stress.

There has been little progress in the overall risk stratification, prevention, and treatment of AKI induced by RM after EHS. AKI is a potential complication of severe rhabdomyolysis, regardless of whether rhabdomyolysis is the result of trauma or some other cause, and the prognosis is substantially worse if renal failure develops. Long-term survival among patients with rhabdomyolysis and AKI is reported to be close to 80%, and the majority of patients with RM-induced AKI recover renal function.^[17]^ Myoglobin, a dark red 17.8-kDa protein, can transport and store oxygen in muscle cells.^[18]^ However, cellular release of myoglobin leads to uncontrolled leakage of ROS, and free radicals cause cellular injury.^[18]^ More recently, it has been shown that myoglobin itself can exhibit peroxidase-like enzyme activity that leads to uncontrolled oxidation of biomolecules, lipid peroxidation, and the generation of isoprostanes.^[19]^ We found that myoglobin levels were predictive of AKI after EHS, independent of relevant clinical characteristics, including baseline kidney function. In addition, the highest myoglobin quartile (*vs*. the lowest, quartile 1) had an adjusted odds ratio of 18.95 (95% confidence interval [CI], 6.00 to 59.83) for AKI, and the OR (*vs*. quartile 2) was 7.92 (95% CI, 1.62 to 38.89) for AKI at discharge and death at 90 d. Improved assessment of the risk of AKI after EHS would allow for more informed decision-making and would help to identify a subgroup of patients who would benefit from an intervention to minimize AKI, potentially in the form of antimyoglobin therapies.

A wide spectrum of clinical contexts in which myoglobin levels are associated with the incidence of EHS-complicated AKI. On the basis of our cell models, we speculate that there may be a synergistic effect between myoglobin, which may enhance the intracellular Fe^2+^ and ROS accumulation of human kidney proximal tubular cells, leading to ferroptosis. Ferroptosis is a new type of cell death that was discovered in recent years and is usually accompanied by a large amount of iron accumulation and lipid peroxidation during the cell death process; the occurrence of ferroptosis is iron-dependent.^[20,21]^ Ferroptosis-inducing factors can directly or indirectly affect glutathione peroxidase through different pathways, resulting in a decrease in antioxidant capacity and accumulation of lipid ROS in cells, ultimately leading to oxidative cell death.^[22,23]^ A lot of evidence proved that ferroptosis is positively regulated by p53, cysteinyl transferase ribonucleic acid synthase (CARS), transferrin receptor (TFR1), SLC7A11 and heat shock protein β1 (HSPβ1), nuclear factor E2 related factor 2 (NRF2), and GPX4.^[24]^ Among the many members of the GPX family, GPX4 plays a pivotal role in the occurrence of ferroptosis and is the key regulator of its occurrence, mainly by inhibiting the formation of lipid peroxides.^[25]^ p53 signaling has also been shown to downregulate the expression of SLC7A11 and affect the activity of GPX4, resulting in the reduction of antioxidant capacity, ROS accumulation, and ferroptosis.^[26, 27, 28]^ Using a model of in vitro heat stress, we found that the expression of p53 was increased and the expression of SLC7A11 and GPX4 was markedly decreased, accompanied by a large amount of Fe^2+^ accumulation and ROS production, in response to myoglobin under heat stress in HK-2 cells. The absence of GPX4 can increase the accumulation of lipid, peroxidation products in renal tubular epithelial cells (such as lipid hydroperoxides, LOOH), and eventually lead to acute renal failure.^[29]^ We confirmed the occurrence of ferroptosis in HK-2 cells induced by myoglobin under heat stress. It is known that if the serum myoglobin is more than 100 mg/dL, there will be tawny urine and apoptosis of renal tubular cells.^[30]^ The concentration of myoglobin (1.5 mg/dL) is just the lowest value serving as the baseline of myoglobinuria (renal threshold), indicating that myoglobin concentration has different effects on renal tubular injury, emphasizing the possibility of ferroptosis in the early stage of AKI.

Mitochondria seem to be involved in ferroptosis induced by cystine deprivation (CDI), which, indeed, is associated with mitochondrial membrane hyperpolarization and lipid peroxide accumulation.^[31]^ In the present study, we found that ERS plays an important role in the process of ferroptosis through the induction of unfolded proteins.^[32]^ ERS, a common stress response in eukaryotic cells, is regulated by multiple factors, including the inositol requiring enzyme 1 (IRE1)-X-box binding protein 1 (XBP1)/TNF receptor associated factor 2 (TRAF2) pathway, ATF6 pathway, and PERK-eukaryotic translation initiation factor-2 (eIF2α)-ATF4 pathway.^[33,34]^ During excessive ERS, unfolded or misfolded proteins continue to accumulate, resulting in the elevation of the expression of proapoptotic transcription factor DNA damage-inducible transcription 3 (DDIT3, CHOP), which induces cell damage.^[35,36]^ ERS plays an important regulatory role in the occurrence and development of many diseases, such as primary glomerulopathy, diabetic nephropathy, drug-related tubulointerstitial injury, renal ischaemia-reperfusion injury, and many other kidney diseases.^[37,38]^ Our findings firmly establish that myoglobin aggravates the ERS response in HK-2 cells during heatstroke, as shown by the elevated expression of p-PER/PERK, p-eIF2, p-IRE1/IRE1, ATF-4, ATF-6, and CHOP. Of note, the ERS response is closely related to ferroptosis, whose agonist can synchronously activate the ERS response in cancer cells, and the activation of the ERS pathway inhibits ferroptosis and leads to drug resistance in cancer cells.^[39,40]^ In some pathological conditions, the activation of the ERS pathway through the induction of unfolded proteins exacerbates the occurrence of ferroptosis.^[41,42]^ Additionally, ferroptosis could promote the cystine–glutamate antiporter system Xc^-^, which leads to ERS.^[43,44]^ Whether ERS is involved in the process of ferroptosis induced by myoglobin under heat stress is unknown. Therefore, the mechanism was verified in HK-2 cells in detail. In the in vitro experiments, it was demonstrated that attenuating ferroptosis by an ERS inhibitor could directly increase cell viability and GPX4 and SLC7A11 levels, decrease p53 activation, and decrease ROS and intracellular Fe^2+^ in response to myoglobin after heat stress, indicating that myoglobin modulated HK-2 cell ferroptosis involving the ERS response after heat stress.

Although some progress has been made in the treatment of RM complicated with AKI after heatstroke, uncovering new therapeutic targets for the prevention of AKI following heatstroke complicated with RM is important. Based on the above observations, we further determined whether drugs associated with inhibition of ERS or ferroptosis would be useful for the treatment of AKI after heatstroke complicated with RM. Deferoxamine can inhibit lipid peroxidation, reduce the ferrous form of myoglobin, and alleviate RM and renal injury in rats, but it has a certain toxicity to the kidney due to the hydrophilicity of deferoxamine. Deferoxamine oral substitutes for α-hydroxypyridines, such as desferridone, have been confirmed by a large number of clinical trials and can be used in patients with high ferrous load after repeated blood transfusion. However, no studies have shown that deferrone can effectively prevent or treat AKI caused by RM. Baicalein is a plant-derived flavonoid that has antioxidant, antiapoptotic, and anti-inflammatory effects in a variety of diseases.^[45,46]^ Through the multidimensional multiinformation (md-mi) xanthine oxidase and superoxide anion fingerprint, baicalein mainly inhibits xanthine oxidase (XOD), and baicalein produced in Gansu Province in China has the best activity in both inhibiting XOD activity and scavenging ROS and regulating different cell deaths by inhibiting ROS.^[47]^ Baicalein can promote the functional recovery of spinal cord ischaemia-reperfusion injury by inhibiting focal death, reducing ERS-mediated apoptosis and activating autophagy. Baicalein can also block arachidonic acid 12 lipoxygenase (ALOX12), which is necessary for p53 to mediate tumor inhibition through different ferroptosis pathways.^[48,49]^ This experiment found that baicalein played a vital role in the process of myoglobin-mediated renal tubular injury after heat stress and effectively relieved myoglobin-mediated renal tubular injury by inhibiting ERS and ferroptosis.

## Conclusions

Serum myoglobin was associated with incident AKI induced by RM after EHS. Serum myoglobin ≥ 1000 ng/mL can predict the occurrence and 90-day prognosis of AKI in EHS. The experimental models used here suggest that myoglobin may be a pathogenic factor in human kidney proximal tubular cells after heat stress by aggravating cell ferroptosis and the ERS response. Baicalein might be a targeted therapeutic drug to reduce AKI.
